# Comparison of left and right ventricular dimensions, systolic and diastolic function between 1.0T Open MRI and 1.5T cylindrical MRI

**DOI:** 10.1186/1532-429X-14-S1-P153

**Published:** 2012-02-01

**Authors:** Philip G Petry, Nina P Hofmann, Florian Andre, Henning Steen

**Affiliations:** 1 Department of Cardiology, University of Heidelberg, Heidelberg, Germany; 2 Facharztpraxis für diagnostische Radiologie Philip G. Petry, Heidelberg, Germany

## Background

Cardiac MRI is the reference standard for cardiac morphology and functional assessment of left and right ventricular (LV,RV) end-diastolic and -systolic volumes (EDV, ESV), stroke volume (SV), ejection fraction (EF) and myocardial mass (Mass) due to its high image resolution tissue differentiation. Because of its cylindrical shape and resulting spatial restrictions, a significant amount of obese and claustrophobic patients, at least 10-15%, cannot be studied at all or only with higher amounts of sedation. Recently, 1.0T open MRI, which offers more space due to its different magnet architecture, has been introduced mainly for orthopaedic purposes but until now has rarely been used for cardiovascular functional measurements.

We sought to investigate the performance of 1.0T open MRI for cardiovascular function and to compare LV- and RV-EDV, -ESV, -SV, -EF, longitudinal RV and LV function and Mass for both 1.0 and 1.5T.

## Methods

Eight volunteers (all male, 27±3ys) were scanned twice within 1.5hours randomly both on the 1.5T (Philips 1.5T Achieva) and the 1.0T (Philips 1.0T Panorama high field open). 1.5T and 1.0T cine SSFP (1.5T:TR/TE=3.5/min,Flip-angle=40°, resolution:1.7*1.8*8mm,slice-thickness=8mm,30heart-phases; HFO 1.0T:TR/TE: 4.7/2.2 msec,flip-angle:70°,resolution:1.8×2×8mm3,slice-thickness=8mm, 30 heart phases) were performed to assess LV- and RV-EDV, -ESV, -SV, -EF and Mass. Mitral annular plane systolic excursion (MAPSE) and tricuspid annular plane systolic excursion (TAPSE) where measured comparable to echocardiography. All images were compared by two blinded observers on a workstation (Philips Viewforum). P<0.05 was regarded statistically significant.

## Results

**Figure 1:**
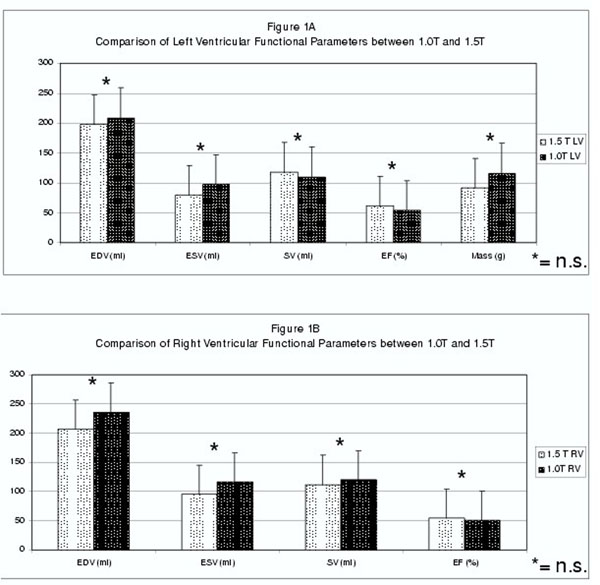


Scans could be successfully performed in all volunteers. Mean breath-hold time for 1.5T was 9sec, for 1.0T 12 sec. At 1.5T, LV-EDV, -ESV and -Mass were insignificantly lower, whereas SV and EF were slightly higher compared to 1.0T (all p=n.s.,figure 1A). For 1.5T, RV-EDV, -ESV and -SV tended to be insignificantly lower, whereas EF was slightly higher compared to 1.0T (all p=n.s.). At 1.5T, MAPSE was 12±1mm, TAPSE was 20±2mm, at 1.0T 12±2 and 22±2mm (for all p=n.s.,figure 1B).

## Conclusions

Measurement of global and longitudinal LV- and RV-function was comparable for 1.5T and 1.0T open. There was a tendency for slightly lower EDV and ESV but higher Mass values at 1.5T, but failed to reach statistical significance. Whether these results are due to the small differences in temporal and spatial resolution at 1.0T or potentially due to the small sample size in these initial experiments warrants further investigation. 1.0T could be a valid clinical alternative for a cardiac MRI assessment in patients with obesity and claustrophobia.

## Funding

None.

